# Whooping-Cough Cases in Children's Hospitals

**Published:** 1898-03-05

**Authors:** 


### The Hospital

A Journal of

The Medical Sciences and Hospital Administration.

Vol. XXIII.—No. 597]. Edited by Sir Henry Burdett, K.C.B., and Solomon C. Smith, M.D., M.R.C.P. [March 5, 1898.

### Whooping-cough Cases in Children's Hospitals.

A question of very considerable importance has lately come under discussion at one of our children's hospitals, namely, whether or not to provide a ward for the reception of patients suffering from whooping-cough.

There can be no doubt that children suffering from this disease are in a somewhat unhappy case. The disease is so definitely infectious that it is impossible, except under pressure of urgent necessity, to admit those affected with it into wards occupied by other cases; yet, on the other hand, it is not a notifiable disease, and is not one of those for which provision is made by the sanitary authorities, so that the unfortunate sufferers are but too apt to fall between two stools. Of the gravity of the malady, and of the very large mortality caused by it, there unfortunately is no question. With the exception of measles and diphtheria, whooping-cough is the most fatal of all the infectious diseases, having been responsible for no less than 6,245 deaths in the 33 large towns of England and Wales during the year 1896, and 4,453 deaths during the year 1897.

On every side of London we see great hospitals erected for the reception, the isolation, and the treatment of scarlet fever, and great is the outcry if a few cases are refused admission. Yet whooping-cough, to which no one seems to give much heed, is just as infectious as scarlet fever, and far more fatal. In fact, the loss of life from whooping-cough in London is in some years more than three times as great as that from scarlet fever.

If this fact is borne in mind, it must seem a strange anomaly that not only is no special provision made for cases of whooping-cough in our great hospitals, but that patients affected by the acute complications which so commonly occur in its course, and are, indeed, the main causes of its high fatality, are, by the very fact that they are suffering from an infectious malady, to a large extent debarred from hospital treatment.

Any systematic attempt to deal effectively with whooping-cough would, however, be a task so great as to stagger even our public bodies, while private charity is probably kept from moving in the matter by the consciousness that whatever it could do would be but a drop in the bucket. And perhaps this would be true if we were to take the view that the object of providing hospital treatment for whooping-cough was to "control" the disease, to limit its spread, and to lessen its epidemics. Whooping-cough, however, stands very much on the same footing as measles in regard to its epidemicity. It is very infectious, it is infectious before its characteristic symptoms show themselves, and its epidemics clearly depend more upon surrounding conditions and the presence of many "susceptibles," than upon the existence of a little more or a little less "infection."

To those who have looked into the matter carefully and have regarded it from the wider point of view, it has become abundantly clear that any attempt to control whooping-cough otherwise than by those sanitary measures by which its fatality is lessened would be wasteful and futile. But the provision of hospital treatment for those complications by which whooping-cough kills is quite another affair, and this more especially applies to those affections which kill by way of the chest. There can be but little doubt that the fatality of the broncho-pneumonia which is so common a cause of death in whooping-cough is due to the infective character of the bronchial catarrh by which it is so often complicated among the poor and in overcrowded and insanitary homes. Diseases of the upper air passages are greatly influenced in their character by the surroundings amid which they occur, and in whooping-cough there is a special tendency for the substance of the lung to become infected with whatever micro-organisms may exist in the tubes that lead to them.

Nothing can be more difficult, and even hopeless, than the attempt to treat the infective broncho-pneumonia of whooping-cough in the midst of the surroundings in which it has arisen, and there are few cases to which treatment in a warm and well-ventilated hospital is more essential. Yet these very cases often have to be refused admission for want of a separate whooping-cough ward. The demand for whooping-cough wards in connection with our general and children's hospitals is not then to be looked upon as part of an attempt to "control" whooping-cough from the point of view of the medical officer of health, nor as undertaking out of private benevolence duties which ought to fall upon the sanitary authorities; it arises from a desire to give to sick children, suffering from various more or less curable diseases, that help which would be given to them without hesitation were it not for the fact that they carry with them the infection of whooping-cough.

We may, however, go further, and say that no children's hospital, and in fact no general hospital, can be considered complete unless it has some means of isolating whooping-cough, for who shall say at what minute that troublesome disease may not arise among its inmates? Whooping-cough may only be Nature's plan for clearing the world of ricketty children. But nature is often cruel while charity is kind.

